# Resolution of Genetic Map Expansion Caused by Excess Heterozygosity in Plant Recombinant Inbred Populations

**DOI:** 10.1534/g3.114.012468

**Published:** 2014-08-15

**Authors:** Sandra K. Truong, Ryan F. McCormick, Daryl T. Morishige, John E. Mullet

**Affiliations:** * Interdisciplinary Program in Genetics, Texas A&M University, College Station, Texas 77843; † Biochemistry & Biophysics Department, Texas A&M University, College Station, Texas 77843

**Keywords:** plant recombinant inbred lines, excess heterozygosity, genetic map construction, genetic map expansion, R/qtl

## Abstract

Recombinant inbred populations of many plant species exhibit more heterozygosity than expected under the Mendelian model of segregation. This segregation distortion causes the overestimation of recombination frequencies and consequent genetic map expansion. Here we build upon existing genetic models of differential zygotic viability to model a heterozygote fitness term and calculate expected genotypic proportions in recombinant inbred populations propagated by selfing. We implement this model using the existing open-source genetic map construction code base for R/qtl to estimate recombination fractions. Finally, we show that accounting for excess heterozygosity in a sorghum recombinant inbred mapping population shrinks the genetic map by 213 cM (a 13% decrease corresponding to 4.26 fewer recombinations per meiosis). More accurate estimates of linkage benefit linkage-based analyses used in the identification and utilization of causal genetic variation.

Linkage maps, or genetic maps, are the relative ordering of and distance between genetic loci in terms of the frequency of recombination between them. Knowledge of the linkage between loci is useful for the identification and use of causal genetic variation using techniques like map-based cloning, marker-assisted selection, and quantitative trait locus (QTL) mapping. Using this linkage information, the genotype of an observable locus (*i.e.*, a marker) can be used to predict the genotype at proximal loci (*e.g.*, a QTL), and the correct relative ordering of markers can be inferred for applications like genome assembly. As such, accurately calculating the linkage between markers is of practical importance.

For some plant species, including maize and pea, discrepancies in recombination frequencies exist between genetic maps calculated using recombination frequency estimates between markers and cytological maps calculated by observing cytological manifestations of recombination events with microscopy (Hall *et al.* 1997a,b; Anderson *et al.* 2003). In general, the genetic maps predict more recombination events per meiosis than the cytological maps observe, and cytological maps are considered to more accurately represent true recombination rates (King *et al.* 2002). Two of the major factors contributing to this disparity include tight double recombination events and segregation distortion found in marker data (Sybenga 1996; Knox and Ellis 2002). Tight double recombinations are observed when an allele is found in a phase opposite to that of alleles from adjacent markers within a relatively short genetic distance (*e.g.*, <5 cM). The source of tight double recombinations is still an open question; they could arise from biological phenomena such as mutations or gene conversions, or they could be (and experiments have shown that they are generally are) genotyping errors (Lincoln and Lander 1992; Dib *et al.* 1996; Broman *et al.* 1998; Broman and Weber 2000). However, the frequency with which they are observed, even with disparate genotyping technologies, suggest that there may be an underlying biological process responsible for some of these tight double recombinations (Sybenga 1996; Broman *et al.* 1998). Regardless, because of the assumptions implicit in genetic map construction, tight double recombinations in marker data greatly expand genetic maps. For the purposes of this work, due to the dramatic map expansion caused by tight double recombinations and our current inability to conclusively identify their origins, we treat tight double recombinations as genotyping errors and set them to missing as commonly practiced in the literature (Lincoln and Lander 1992; Dib *et al.* 1996; Broman *et al.* 1998).

Similarly, segregation distortion is a commonly observed phenomenon and can also affect the estimation of recombination frequencies. Segregation distortion is the observed deviation of a locus from the expected segregation ratio under the model of Mendelian inheritance, and it generally occurs as a consequence of unequal gametic or zygotic fitness (*e.g.*, artificial selection, meiotic drive, etc.), or as a consequence of an error prone marker. Although one solution for distorted markers is their removal, the removal of markers reduces genome coverage, and techniques have been developed to account for distorted markers by (i) integrating repeated observations in multiple populations (Wang *et al.* 2005; Cloutier *et al.* 2012) or (ii) modeling the differential viability of gametes or zygotes (Lorieux *et al.* 1995a,b; Wu *et al.* 2007; Zhu *et al.* 2007; Xu 2008). In addition, information on segregation distortion can be used to aid in the identification of selection and QTL mapping (Xu 2008).

Multiple reports have documented extensive segregation distortion in plant recombinant inbred populations manifesting mostly as excess heterozygosity, and occasionally as reduced heterozygosity (Supporting Information, Table S1) (Knox and Ellis 2002). Although the source of distortion is not conclusively known, it is hypothesized to be the result of a general selective advantage (or disadvantage) of heterozygote genotypes. Despite the prevalence of heterozygosity in plant recombinant inbred lines (RILs), the techniques developed to incorporate distorted markers are not commonly used. In the case of retaining markers based on multiple observations, this technique necessitates multiple RIL populations (Cloutier *et al.* 2012) which may be too high a barrier given some plant generation intervals. Existing methods to model the viability of each genotype differentially treats each marker pair, and so may suffer from the overfitting of large data sets without specific biological models; these have also only been shown for *BC*_1_ and *F*_2_ populations (Lorieux *et al.* 1995a,b; Zhu *et al.* 2007). In general, plant geneticists have constructed genetic maps of *F_t_* populations, where *t* is the generation interval, by fitting observations to the expected genotype frequencies of a Mendelian fixed RIL model that relies on assumptions of complete fixation, no selection, and no mutation; this model is unable to account for proportions of heterozygosity maintained per generation other than 0.5. If the recombinant inbred population is treated as though all loci are fixed (as *t* → ∞), yet more heterozygosity was maintained per generation than expected by Mendelian segregation on the way to fixation, then the recombination frequencies will be artificially overestimated; not accounting for excess heterozygosity underestimates the number of informative meioses that can occur prior to fixation. In addition, treating RIL populations that have not yet reached fixation as fixed RILs results in the loss of genotypic information and makes incorrect assumptions when calculating recombination fractions. Finally, in cases in which the distortion occurs across the entire genome, such as for the sorghum mapping population used here, removal of distorted markers under a Mendelian *F_t_* model would remove the majority of typed markers, causing a dramatic loss of genetic information.

Here, we build off an existing model of differential zygotic viability to incorporate a heterozygosity maintenance term for plant recombinant inbred populations and find a new solution for the genotype probabilities used to calculate recombination frequencies. We incorporate this calculation for expected genotype frequencies to account for different proportions of heterozygosity maintained per generation (other than 0.5) using the open-source genetic map construction code base from R/qtl (Broman *et al.* 2003) and report its efficacy in a simulated RIL population and a sorghum mapping population.

This modeling allows more accurate generation of genetic maps and retention of more genetic information by accounting for the biological phenomenon of differential fitness of heterozygous loci. More accurate estimations of recombination fractions, and thus linkage, will improve the accuracy of methods that use linkage information to detect and use causal genetic variants.

## Materials and Methods

### Derivation and implementation of genetic model

The model for genetic map construction from genetic markers of a population with known pedigree and markers ordered on the basis of physical position with a reference genome is simplified into calculating recombination fractions between pairs of markers. Here we derive the quantitative genetic theory underlying the expected genotypes of a selfed population, *F_t_*, given a proportion of heterozygosity retained that deviates from Mendelian segregation assumptions. To derive these equations we simultaneously extend and incorporate two models: (i) Bulmer’s general solutions for genotype frequencies of self-fertilized populations based on the work of Haldane and Waddington at two linked loci (Haldane and Waddington 1931; Bulmer *et al.* 1980) and (ii) a model for zygotic differential viability, where each genotype is assigned a fitness (that may confer an advantage/disadvantage) in the *F*_2_ progeny (Wu *et al.* 2007; Zhu *et al.* 2007).

#### Genotype frequencies of selfing populations:

Before we model heterozygosity maintenance into estimating genotype frequencies, we will set up the familiar framework used to estimate genotype frequencies in a traditional RIL. Consider two linked loci (or markers) *α* and *β*. Locus *α* has alleles A and a and locus *β* has alleles B and b. Suppose that the initial parental mating was $\frac{\text{AB}}{\text{AB}}\times \frac{\text{ab}}{\text{ab}}$, then in subsequent generations, *F_t_* where *t* ∈ ℕ is the generation interval, the family of individuals will contain a distribution of ten different genotypes, and for the initial condition of *t* = 1, all genotypes in the *F*_1_ generation ∈ $\frac{\text{AB}}{\text{ab}}$ genotype. Furthermore, due to the symmetry of genotypes under self-fertilization, the genotype probabilities are reduced into $p_{F_{t}}$ as five genotype classes (class *i*, *i* ∈ [1, 2, 3, 4, 5]) as described by Haldane and Waddington (1931), where $\forall \;t,\left\|p_{F_{t}}\right\|=1$.

$$p_{F_{t}}={\left(\begin{array}{c} p\left(\text{class}\;1\right)\\ p\left(\text{class}\;2\right)\\ p\left(\text{class}\;3\right)\\ p\left(\text{class}\;4\right)\\ p\left(\text{class}\;5\right) \end{array}\right)}_{t}={\left(\begin{array}{c} p\left(\frac{\text{AB}}{\text{AB}}\right)+p\left(\frac{\text{ab}}{\text{ab}}\right)\\ p\left(\frac{\text{Ab}}{\text{Ab}}\right)+p\left(\frac{\text{aB}}{\text{aB}}\right)\\ p\left(\frac{\text{AB}}{\text{aB}}\right)+p\left(\frac{\text{Ab}}{\text{ab}}\right)+p\left(\frac{\text{AB}}{\text{Ab}}\right)+p\left(\frac{\text{aB}}{\text{ab}}\right)\\ p\left(\frac{\text{AB}}{\text{ab}}\right)\\ p\left(\frac{\text{Ab}}{\text{aB}}\right) \end{array}\right)}_{t}$$

The transition from one class to another each generation is a Markov chain and is described through the transition probability matrix, **T**, that takes into consideration the gametic outputs of each class for each meiosis event, such that for generation *t* and the initial condition $p_{F_{1}}^{\prime }=\left[0,0,0,1,0\right]$ we satisfy

$$p_{F_{t+1}}^{\prime }=Tp_{F_{t}}^{\prime }$$ (1)

Thus far, we’ve introduced the common modeling of genotype probabilities for the case of self-fertilized populations. Solving for the genotype probabilities is dependent on defining the transition probability matrix, **T**. Under the assumption of Mendelian segregation, **T** is defined and so $p_{F_{t}}$ has a general solution which has been implemented (Bulmer *et al.* 1980; Lander *et al.* 1987; Broman *et al.* 2003). Furthermore, for the case of differential zygotic viability, **T** has been modeled and solved for an *F*_2_ (Zhu *et al.* 2007).

#### Modeling heterozygosity:

Here we model a heterozygosity maintenance term for selfed recombinant inbred populations to account for viabilities of heterozygote genotypes that deviate from Mendelian segregation (where *h* = 0.5). To construct **T** that accounts for the proportion of heterozygosity maintained each generation, *h*, we will examine each class’ expected transition from generation *t* to generation *t* + 1 under a potential deviation of *h* from 0.5.

##### Transition from class 1 and class 2 is fixed:

Class 1 and 2 are the ultimate absorption states as *t* → ∞. For example, the probability of class *i* ≠ 1 in generation *t* + 1 given that the marker pair was in class 1 in generation *t* is zero. Once a marker pair is in either class 1 or class 2, it will remain there.

##### Transition from class 3 depends on h:

Class 3 requires consideration of the segregation of only one marker that is heterozygous in generation *t* as the other marker will be homozygous and thus fixed in any subsequent generation after *t*.

Let $H_{F_{t}}$ be the proportion of heterozygosity observed for all markers of an *F_t_* family and assume that the amount of heterozygosity maintained in all markers, *h*, is constant each generation. Then we can solve for *h* through the following relationship $h^{t-1}=H_{F_{t}}$. *h* will be modeled into the transition probability matrix as a modifier of expected segregation. For data $H_{F_{t}}$,

$$h=e^{\frac{\ln\left(H_{F_{t}}\right)}{t-1}}.$$ (2)

For the heterozygous marker *α* and *h* (in class 3) in generation *F_t_*, the genotype probabilities for generation *F_t_*_+1_ will be dependent on the expected segregation of the alleles of marker *α*, class 1: 2: 3, which is $\frac{1-h}{2}:\frac{1-h}{2}:h$.

##### Transition from class 4 and 5 depends on h and r:

Class 4 and 5 requires consideration of the segregation of two markers that are heterozygous at generation *t* and the recombination frequency, *r*, between the two markers.

Similar to treatment of heterozygosity for one marker, we now apply the same heterozygosity term to both markers *α* and *β*. We model this within the context of zygotic differential viability, as shown in (Lorieux *et al.* 1995b; Wu *et al.* 2007). Assume for marker *α* that the viability of genotype Aa relative to AA or aa is *u* and the same *u* applies to the alleles of marker *β*. Then, the genotype probabilities in generation *F_t_*_+1_ from class 4, $\frac{\text{AB}}{\text{ab}}$, or class 5, $\frac{\text{Ab}}{\text{aB}}$, in generation *F_t_* is dependent on the segregation of alleles of marker *α* and *β*, class 1 : 2 : 3 : 4 : 5. From class 4 this ratio will be $\frac{2{\left(1-r\right)}^{2}}{d}$ : $\frac{2r^{2}}{d}$ : $\frac{8ur\left(1-r\right)}{d}$ : $\frac{2u^{2}{\left(1-r\right)}^{2}}{d}$ : $\frac{2u^{2}r^{2}}{d}$, and from class 5 this ratio will be $\frac{2r^{2}}{d}$ : $\frac{2{\left(1-r\right)}^{2}}{d}$ : $\frac{8ur\left(1-r\right)}{d}$ : $\frac{2u^{2}r^{2}}{d}$ : $\frac{2u^{2}{\left(1-r\right)}^{2}}{d}$, where $d=2{\left(1-r\right)}^{2}+8ur\left(1-r\right)+2r^{2}+2u^{2}\left[{\left(1-r\right)}^{2}+r^{2}\right]$.

To model the amount of heterozygosity retained in generation *t* for a marker pair of class *j*, for *j* ∈ [4, 5], in the previous generation *t* − 1 we model *h*, calculated by equation 2 as

$$h=\frac{1}{2}p\left(\text{class}\;3{\;}_{t+1}|\text{class}\;j{\;}_{t}\right)+p\left(\text{class}\;4{\;}_{t+1}|\text{class}\;j{\;}_{t}\right)+p\left(\text{class}\;5{\;}_{t+1}|\text{class}\;j{\;}_{t}\right)$$ (3)

such that we can calculate *u* with variable *r* and subsequently *d*.

##### Transition probability matrix, **T:**

Incorporating the transition from a given class to all classes in every generation, we now have a transition probability matrix,

$$\begin{array}{l} \;\;\;\;\;\begin{array}{ccccc} \text{class}1{\;}_{t} & \text{class}2{\;}_{t} & \text{class}3{\;}_{t} & \text{class}4{\;}_{t} & \text{class}5{\;}_{t} \end{array}\\ T=\begin{array}{c} \text{class}\;1{\;}_{t+1}\\ \text{class}\;2{\;}_{t+1}\\ \text{class}\;3{\;}_{t+1}\\ \text{class}\;4{\;}_{t+1}\\ \text{class}\;5{\;}_{t+1} \end{array}\left(\begin{array}{ccccc} 1 & 0 & \frac{1-h}{2} & \frac{2{\left(1-r\right)}^{2}}{d} & \frac{2r^{2}}{d}\\ 0 & 1 & \frac{1-h}{2} & \frac{2r^{2}}{d} & \frac{2{\left(1-r\right)}^{2}}{d}\\ 0 & 0 & h & \frac{8ur\left(1-r\right)}{d} & \frac{8ur\left(1-r\right)}{d}\\ 0 & 0 & 0 & \frac{2u^{2}{\left(1-r\right)}^{2}}{d} & \frac{2u^{2}r^{2}}{d}\\ 0 & 0 & 0 & \frac{2u^{2}r^{2}}{d} & \frac{2u^{2}{\left(1-r\right)}^{2}}{d} \end{array}\right) \end{array}$$ (4)

With **T** defined, we solve for the general solution of $p_{F_{t}}$ by equation 1 and initial condition $p_{F_{1}}^{\prime }=\left[0,0,0,1,0\right]$ and use $p_{F_{t}}$ to fit recombination fractions (see Supplemental Materials for calculations and solution). When the expectations of segregation are in fact Mendelian, *h* = 0.5, then as expected the solution for genotype frequencies will reduce to the same ones solved for by Haldane and Waddington (1931).

#### Genotype frequencies with heterozygosity model:

Given the theory derived for $p_{F_{t}}^{\prime }=Tp_{F_{t-1}}^{\prime }$ that is altered with a heterozygosity model, we solved for the general solution of $p_{F_{t}}$, genotype frequencies, using Matlab (2010), and an M-file is provided in File S1 to document all variables defined and calculations.

#### Implementation and simulation:

Calculations of the genotype frequencies for proportions of heterozygosity maintained, *h*, other than 0.5 were implemented in C within a fork of the R/qtl v1.28.19 code base (Broman *et al.* 2003). Specifically we used the golden section search algorithm as implemented in the R/qtl *BC_s_F_t_* tools (Shannon *et al.* 2013) to estimate recombination fractions given genotype data for a marker pair. Map distances were calculated using the Haldane mapping function given the recombination fractions estimated from the golden section search.

The source code is available on GitHub as a forked R/qtl repository at https://github.com/MulletLab/qtl. The hetexp branch contains the new functions, including est.rf.exHet() that can be called from R similar to the existing est.rf() but with a heterozygosity term, *h*, passed to it. The est.rf.exHet() function can also estimate *h* on the basis of H for each linkage group. Example usage can be found at https://github.com/MulletLab/exHet_Supplement.

Genotypes for a 200-cM linkage group genotyped for 1000 individuals at 1000 markers were simulated under the derived heterozygosity model both (i) without errors or missing data, and (ii) with 1% errors and 5% missing data. The code used to generate the datasets, the simulated datasets, and their respective results can be found at https://github.com/MulletLab/exHet_Supplement.

### Plant materials and genotyping

The sorghum recombinant inbred mapping population, BT×623 × IS3620C, were made available by the USDA-ARS Plant Genetic Resource and Conservation Unit, Griffin, GA (Burow *et al.* 2011). These *F*_7–9_ individuals were planted in fields in College Station, TX, in the summer of 2013. DNA was extracted from leaf tissue of 10−12 plants from seed stock of each RIL and prepared by digital genotyping with restriction endonuclease *NgoMIV* (Morishige *et al.* 2013). The digital genotyping templates were sequenced on Illumina HiSequation 2500 with 72 (or fewer) samples per lane.

Genotypes were generated from the sequenced reads of the recombinant inbred lines and their parents, BT×623 and IS3620C. The sequence reads were delivered already sorted on sample barcode, and they were checked for restriction sites using awk; where applicable, preprocessing was parallelized using GNU parallel (Tange 2011). Reads were aligned to the sorghum reference genome (Sbi1) with BWA mem (v 0.7.5a) (Paterson *et al.* 2009; Li and Durbin 2010). Aligned reads were realigned around indels using the Genome Analysis Toolkit (GATK v3.1-1) and the Queue framework with IndelRealigner; individual GVCFs were generated using the HaplotypeCaller; and joint genotyping was performed using GenotypeGVCFs (McKenna *et al.* 2010; Depristo *et al.* 2011; Van Der Auwera *et al.* 2013). Variants were hard filtered using VariantFiltration under the following criteria: DP < 10; QD < 5.0; MQ < 30.0; MQRankSum < ×10.0; BaseQRankSum < −10.0. The remaining variants were filtered to keep only biallelic variants for which the two parents, BT×623 and IS3620C, were each homozygous for different alleles and to keep only variants that were genotyped with a GQ score ≥ 20 in ≥ 25% of the samples. For these genotypes, the median depth of reads that passed the HaplotypeCaller’s internal quality control metrics (*i.e.*, the median sample-level DP annotation) was 17 reads. Genotypes with a GQ score <20 were set to missing, and those remaining were screened for tight double recombinations occurring within 2 kbp; genotypes involved in a tight double recombination were set to missing. These variants and genotypes were used as the initial input for genetic map construction in R/qtl.

### Genetic map construction

Genetic map construction was performed as an iterative process in R/qtl, starting with 424 individuals (RILs) genotyped at 12,836 single-nucleotide polymorphisms and indel markers. Two individuals and 1340 markers were removed due to high missingness levels (≥60%), seven individuals were removed due to sharing ≥90% of genotypes with another individual, 703 markers were removed for being uninformative due to close proximity, and 17 individuals were removed for having genotypic proportions far outside the distribution of most of the population members. The remaining 398 individuals and 10,793 markers had an overall 7.4% heterozygous genotypes. The percentage 7.4% was used as an initial *H*_0_ to test for segregation distortion. Markers that deviated largely from a 463:74:463 ratio (homozygous parent 1:heterozygous:homozygous parent 2) by a χ^2^ test (*P* < 1 × 10^−15^) were excluded; due to extreme segregation distortion for one parental allele on chromosome 1 caused by artificial selection of a known flowering time QTL, we dramatically relaxed the distortion threshold (*P* < 1 × 10^−30^) for chromosome 1, although a large gap still remains due to failing to retain markers in the region of the most severe distortion (Yang *et al.* 2014). After filtration, 398 individuals and 10,090 markers remained with 7.5% heterozygous genotypes. 7.5% was considered to better represent the true percentage of heterozygous genotypes in the dataset, and so we applied the segregation distortion test for the 10,793 markers with the updated *H*_1_ of 7.5%; this retained 398 individuals typed at 10,091 markers. Of note, at our *P*-value thresholds, the expected Mendelian ratio (*H* = 1.6%) retained only 4512 markers, whereas the excess heterozygosity model (*H* = 7.5%) retained 10,091 markers.

With the 398 individuals typed at 10,091 markers, we then constructed an initial genetic map by estimating recombination fractions calculated under the excess heterozygosity model and R/qtl’s implemented Haldane mapping function with markers grouped and ordered by their physical position on the Sbi1 reference genome. Ten markers on chromosome 6 were removed due to their incorrect placement on the Sbi1 reference assembly, as indicated by inspection of recombination fractions and previous work (Morishige *et al.* 2013). The genetic map was then re-estimated, and tight double recombinations less than or equal to 2.0 cM were removed. The proportion of heterozygosity at this point, *H* = 6.7%, was used to estimate *h* for use in the final map estimation under the excess heterozygosity model; the same markers and genotypes were used for map estimation under the Mendelian model. Genetic maps were estimated directly from calculated pairwise recombination fractions for adjacent markers using R/qtl’s implemented est.rf() and our implemented est.rf.exHet().

## Results

### Excess heterozygosity generally causes overestimation of recombination frequencies

**Figure 1:**
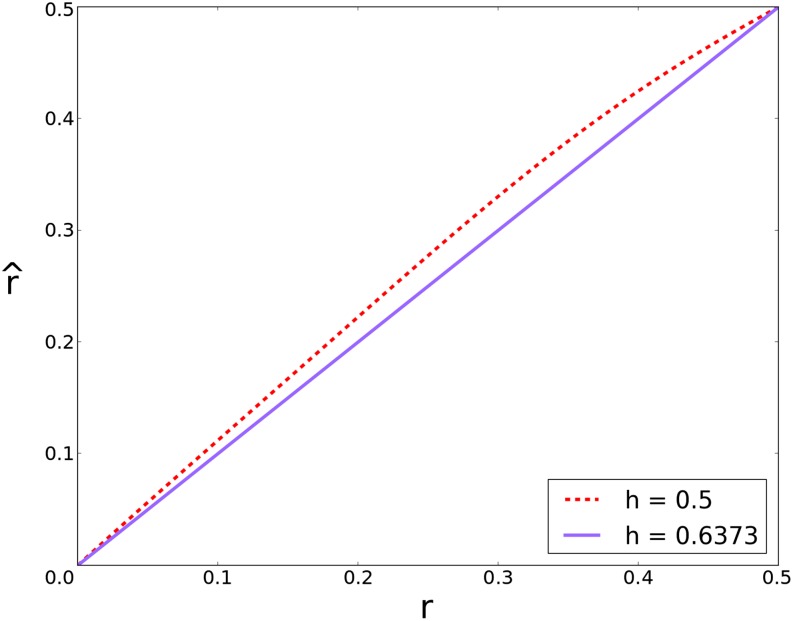
Estimated recombination frequencies, $\hat{r}$, under excess heterozygosity and Mendelian models. Recombination frequencies estimated from genotype frequencies under Mendelian expectations (*h* = 0.5) *vs.* under modeling a global heterozygosity advantage (*h* = 0.6373) at generation *t* = 7 of a selfing population. This shows that if the population was retaining excess heterozygosity (at a rate of 63.73% each generation as opposed to the Mendelian 50%), then estimating recombination fractions under Mendelian expectations would lead to overestimation of the recombination frequency underlying the data and subsequent map expansion.

To demonstrate how excess heterozygosity expands genetic maps, we plotted the estimated recombination frequency, $\hat{r}$, given genotype counts expected under conditions of excess heterozygosity for different recombination frequencies, *r*, estimated using the Mendelian model and using the derived heterozygosity model (see the section *Materials and Methods* for the derivation and implementation). When the genotype counts for the two markers arise from an excess heterozygosity model for an *F*_7_ RIL population, accounting for the excess heterozygosity when calculating $\hat{r}$ correctly estimates the recombination frequency, *r*, underlying the data (Figure 1). However, use of the Mendelian model to estimate $\hat{r}$ results in an overestimation relative to the recombination frequency underlying the data. Overestimation of $\hat{r}$ decreases as linkage increases (*r* ≤ 0.3), and even these small overestimations between many pairs of markers lead to map expansion proportional to the genetic distance of the region with excess heterozygosity.

To further demonstrate the effects of excess heterozygosity, we simulated an *F*_7_ RIL population of 1000 individuals with a 200-cM linkage group covered by 1000 markers under conditions of excess heterozygosity maintained per generation (*h* = 0.6373). Estimating recombination frequencies under a Mendelian model (*h* = 0.5) overestimates the map by 18.0% (236.0 cM), whereas accounting for excess heterozygosity in the genetic model yields a genetic map that differs from the simulated distance by only 2.5% (204.9 cM) (Figure S5).

### Incorporation of a heterozygosity term into the genetic model shrinks a sorghum genetic map

**Figure 2:**
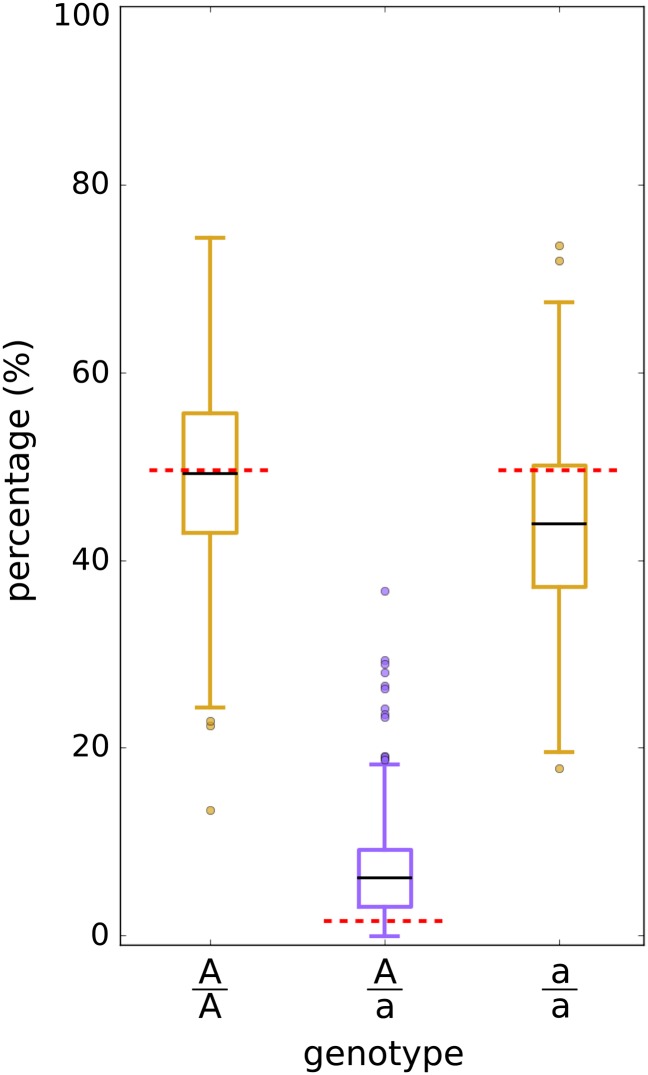
Excess heterozygosity in a sorghum mapping population. Box plot of genotype frequencies of 398 individuals of the BT×623 × IS3620C recombinant inbred population. Each individual has a percentage of its genotypes that are homozygous or heterozygous for a BT×623 parental allele, A, and IS3620C parental allele, a. The dashed red lines represent the expected genotype frequencies under the assumptions of Mendelian segregation. The expected heterozygous frequency is lower than the median observed.

**Figure 3:**
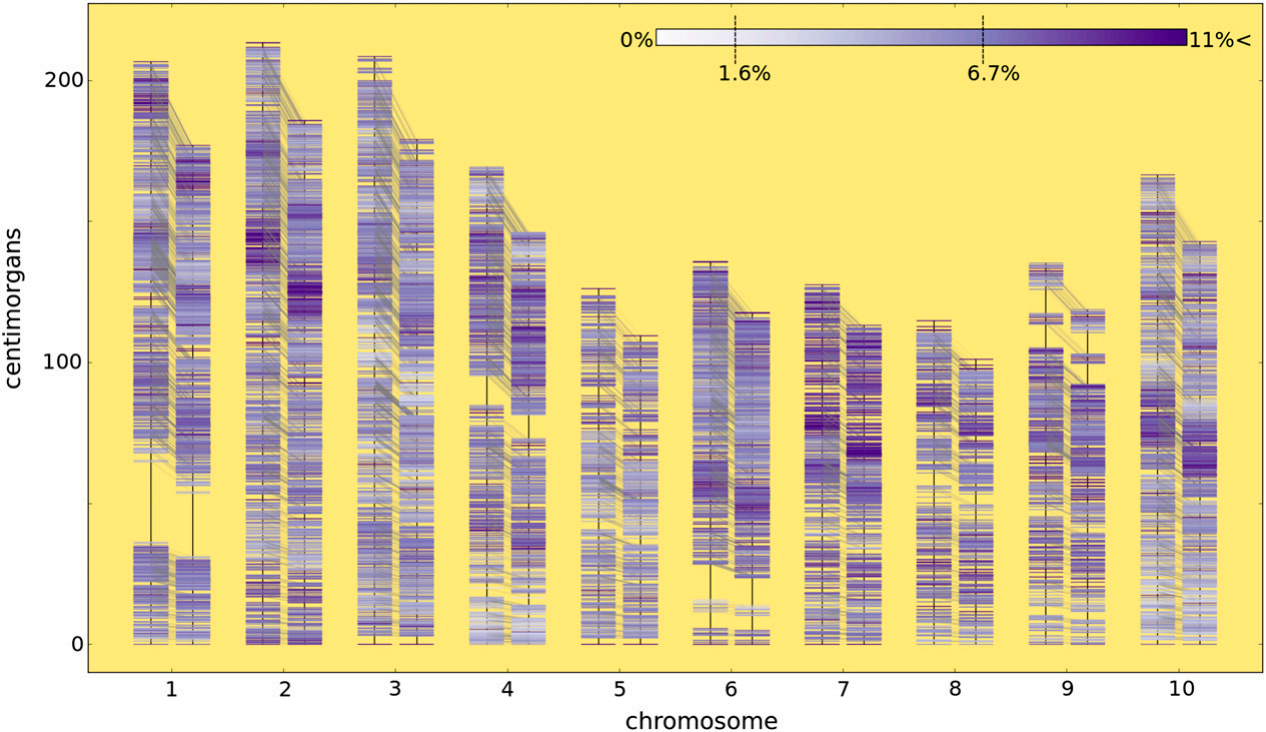
Accounting for excess heterozygosity shrinks the sorghum genetic map. This plot shows the genetic position of 10,081 markers for two genetic maps. For each chromosome, the genetic map on the left is calculated under the Mendelian segregation model. The genetic map on the right is calculated under the excess heterozygosity model. For all chromosomes (#1−10), the map shrinks by accounting for excess heterozygosity. The coloring of the markers correspond to the percentage of heterozygosity at that locus (no heterozygosity, white, to high (>11%) heterozygosity, purple). The expected heterozygosity of an *F*_7_ RIL population is 1.6% and the observed heterozygosity in the BT×623 × IS3620C population was 6.7% as depicted on the color bar. Faint gray lines connect a marker’s position in one map with its corresponding position in the other map.

To demonstrate that accounting for excess heterozygosity can shrink the genetic map of a plant recombinant inbred population (as postulated by Knox and Ellis 2002), we applied our method to a sorghum recombinant inbred population displaying excess heterozygosity (Burow *et al.* 2011). The members of the population ranged from *F*_7_ to *F*_9_ and exhibit more than a 300% increase in heterozygosity relative to the expected heterozygosity given a Mendelian model: 6.7% observed after our quality control steps *vs.* 1.6% given a Mendelian model for *t* = 7 (Figure 2). Heterozygosity was present at elevated levels throughout the genome relative to expectations under a Mendelian model, although some regions deviated notably from the average (Figure 3, Figure S2, and Figure S3). Previous reports estimating the genetic map as an RIL that has gone to fixation for this sorghum population range from 1279 cM to 1713 cM, a difference of 8.48 recombinations per meiosis (Table S2) (Peng *et al.* 1999; Hart *et al.* 2001; Menz *et al.* 2002; Mace *et al.* 2009; Burow *et al.* 2011).

The genotype calls for this population were used to parameterize the heterozygosity term, *h*, by treating the population as an *F*_7_ such that $H_{F_{7}}$= 0.067 and *h* = 0.6373 by equation 2; *u* and *d* were subsequently found by equation 3 (*Materials and Methods*). Figure 3 compares the genetic maps of the sorghum recombinant inbred population estimated as an *F*_7_ under Mendelian expectations (*h* = 0.5) on the left and estimated under the excess heterozygosity model (*h* = 0.6373) on the right for each chromosome. Once excess heterozygosity is accounted for, the genetic map shrinks from 1603.8 cM to 1390.6 cM, a 213.2-cM difference corresponding to a 13% decrease, or 4.26 recombinations fewer recombinations per meiosis. As expected, the derived heterozygosity model behaves identically to the Mendelian model when *h* = 0.5 (Table 1).

**Table 1:** Genetic maps estimated from the BT×623 × IS3620C sorghum recombinant inbred mapping population

Chr	est.rf()	est.rf.exHet (*h* = 0.5)	est.rf.exHet (*h* = 0.6373)	Burow *et al.* (2011)
1	206.7	206.7	177.0	231.6
2	213.4	213.4	185.9	205.0
3	208.6	208.6	179.2	202.4
4	169.2	169.2	146.2	174.4
5	126.2	126.2	109.4	138.2
6	135.7	135.7	117.6	115.6
7	127.5	127.5	113.0	155.7
8	114.7	114.7	101.0	152.3
9	135.3	135.3	118.7	153.0
10	166.6	166.6	142.8	148.4
Total	1603.8	1603.8	1390.6	1676.6

Except for the map reported by Burow *et al.* (2011) (which was treated as a fixed RIL), maps were estimated as a selfed F7 population. The est.rf() function uses R/qtl’s native recombination frequency calculations, whereas est.rf.exHet() uses the calculations detailed in the section *Materials and Methods* with the respective h values. The map produced by Burow *et al.* (2011) from a subset of the BT×623 × IS3620C population is provided as reference. RIL, recombinant inbred line.

## Discussion

Observations that deviate from a model’s expectations, such as segregation distortion caused by excess heterozygosity, will generally cause the model to generate inaccurate estimations; unsurprisingly, excess heterozygosity leads to unexpected map lengths when the genetic map is estimated under the assumptions of Mendelian segregation. Here we have shown that the excess heterozygosity present in a sorghum recombinant inbred population caused map expansion under Mendelian expectations. However there is no theoretical reason why excess heterozygosity could not also shrink the genetic map under certain conditions. If we had observed this recombinant inbred population in its *F*_3_ stage and parametrized an *h* = 0.6373 (the same amount of heterozygosity maintained each generation), excess heterozygosity in this case would cause recombination frequencies to be underestimated under the Mendelian model (Figure S1). Although this result does not agree with the idea that excess heterozygosity always causes map expansion (Knox and Ellis 2002), it is not an unexpected one; our assumptions are dependent on a RIL approaching fixation (*t* → ∞), in which case the longer maintenance of heterozygous loci provides more opportunities for recombination at the given loci. In other words, in the context of the genetic model derived in the *Materials and Methods*, as *t* → ∞, the proportion of class 2 genotypes ($\frac{\text{Ab}}{\text{Ab}}$ and $\frac{\text{aB}}{\text{aB}}$) will be larger for populations with excess heterozygosity than those following Mendelian expectations. Under our model and moderate values of *h* (*e.g.*, *h* = 0.6373), excess heterozygosity is predicted to cause map shrinkage for small generation values (*e.g.*, *t* = 3), and map expansion for larger generation values (*e.g.*, *t* = 7). The general case is simply that, when the observed genotype frequencies deviate from those predicted by the model, the estimated recombination fractions, $\hat{r}$, will be inaccurate.

Our modeling was done under the assumption that the amount of heterozygosity maintained by each generation is evenly distributed among markers. While our data for this population show that excess heterozygosity is present throughout the genome, there is also local variation (Figure 3 and Figure S2). This finding agrees with previous work showing that hybrid advantage and/or disadvantage can localize to specific loci in the genome (Li *et al.* 1997), and in these cases it may be more appropriate to obtain an *h*_marker pair_ from data $H_{F_{t}}$ for each marker pair (which we derive in File S1). However we chose not to implement this method for our mapping population to avoid overfitting the data and to maintain an expected global value with which the genetic map could be curated. We have additionally implemented an option to parameterize *h* for each linkage group based on the *H* of the genotypes for the linkage group, though this yielded little difference for our use case (Figure S5). Ultimately, our solution strikes a balance between an *a priori* model based on Mendelian segregation and parameterizing the model based entirely on each marker pair. Future work may explore an intermediate approach based on estimating regional heterozygosity levels to paramaterize *h* for groups of markers; an examination of the biological mechanisms underlying these regional deviations from the global level of maintained heterozygosity is also merited (Figure S2, Figure S3, and Figure S4).

We have made the implementation of this method available as a fork of the R/qtl code base at https://github.com/MulletLab/qtl, and provide examples for its use at https://github.com/MulletLab/exHet_Supplement so that it can be used where appropriate for conditions of excess heterozygosity; we are grateful to the R/qtl developers for making their code base accessible to be built upon (Broman *et al.* 2003). We believe this work serves as an example of when to extend a genetic model to fit observations of biological phenomena that deviate from traditional expectations, and that the differential zygotic viability model (Zhu *et al.* 2007) will serve as a useful base to modify as the mechanisms underlying segregation distortion become better understood. As genotyping technologies continue to improve, so too should the models we use to interpret the phenomena underlying the data. Deviations from traditional models, including segregation distortion and tight double recombinations, will need to be corrected to generate genetic maps that have reasonable agreement with the cytological maps calculated using microscopy to observe indicators of recombination events. More accurate genetic maps will improve linkage based analyses such as map-based cloning, marker-assisted selection, and QTL mapping, as well as assist marker ordering for genome assembly and provide better estimates of how recombination is distributed in the genome.

## Supplementary Material

Supporting Information
