# Supplementary material for: Resolution of Genetic Map Expansion Caused by Excess Heterozygosity in Plant Recombinant Inbred Populations
Source: G3 (Bethesda). 2014 Aug 15;4(10):1963–9. doi: 10.1534/g3.114.012468 (PMC4199702; doi:10.1534/g3.114.012468)
Supplement: Supporting Information [file supp_4_10_1963__index.html]

Resolution of Genetic Map Expansion Caused by Excess Heterozygosity in Plant Recombinant Inbred Populations — Supporting Information 

# Resolution of Genetic Map Expansion Caused by Excess Heterozygosity in Plant Recombinant Inbred Populations

## Supporting Information for Truong *et al.*, 2014

**Files in this Data Supplement:**

- File S1 - Supporting Text, Figures S1-S5, and Tables S1 and S2 (PDF, 1.6 MB)
- Figure S1 - Estimated recombination fractions, ^r, of excess heterozygosity versus Mendelian expectations for *t* = 3 (PDF, 120 KB)
- Figure S2 - Heterozygosity landscape (PDF, 468 KB)
- Figure S3 - Heterozygosity distribution in sorghum F7 mapping population (PDF, 784 KB)
- Figure S4 - Heterozygosity on chromosome 2 (PDF, 329 KB)
- Figure S5 - Screenshot of spreadsheet containing map estimation results for the sorghum mapping population and simulated data with different models and methods (PDF, 414 KB)
- Table S1 - Reports of deviation from expected heterozygosity maintained per generation (PDF, 363 KB)
- Table S2 - Reported genetic map sizes for the sorghum BTx623 x IS3620c RIL population used in this study (PDF, 271 KB)
