# Supplementary material for: Resolution of Genetic Map Expansion Caused by Excess Heterozygosity in Plant Recombinant Inbred Populations
Source: G3 (Bethesda). 2014 Aug 15;4(10):1963–9. doi: 10.1534/g3.114.012468 (PMC4199702; doi:10.1534/g3.114.012468)
Supplement: Supporting Information [file supp_g3.114.012468_FileS1.pdf]

Supplemental Information

Resolution of genetic map expansion caused by excess heterozygosity in plant recombinant inbred populations

Sandra K. Truong[1,2,†], Ryan F. McCormick[1,2,†], Daryl T. Morishige[2], John E. Mullet[1,2,*]

1 Interdisciplinary Program in Genetics, Texas A&M University, College Station, Texas, United States of America
2 Biochemistry & Biophysics Department, Texas A&M University, College Station, Texas, United States of America
† These authors contributed equally.
∗ Corresponding author email: jmullet@tamu.edu

CONTENTS

Solving for the general solution, p_{F_t}, in MATLAB **7 SI**

Parameterization of the heterozygosity term **11 SI**
A global heterozygosity term . . . 11 SI
Local heterozygosity term for each linkage group, $h_{\text{linkage group \#}}$ . . . 11 SI
The derivation for a local heterozygosity term for each marker, h_{marker} 13 SI

Genetic map estimations of simulated datasets with excess heterozygosity **17 SI**
Tight double recombinations . . . 18 SI
Error probability in the hidden Markov model . . . 19 SI
F7 versus fixed RIL ($t \to \infty$) . . . 19 SI

LIST OF FIGURES

S1 **Estimated recombination fractions, $\hat{r}$, of excess heterozygosity versus Mendelian expectations for $t = 3$.** Recombination fractions estimated from genotype frequencies under Mendelian expectations (h=0.5) versus under modeling a global heterozygosity advantage (h=0.6373) at generation $t = 3$ of a selfing population. This shows that if the population was retaining excess heterozygosity (at a rate of 63.73% each generation as opposed to the Mendelian 50%), then estimating recombination fractions under Mendelian expectations would shrink the map if observed at generation $t = 3$. . . . . . 6 SI

S2 **Heterozygosity landscape.** Dot plot of the proportion of heterozygous genotypes versus the physical base pair position of the 10,081 markers. The coloring of the markers correspond to the percentage of heterozygosity as explained in Figure 3. The Mendelian expected proportion of heterozygosity of an F_7 RIL population is 0.016 and the observed heterozygosity as an average of the BTx623×IS3620c F_7 is 0.067 depicted by a red dashed line and purple solid line, respectively. . . . . 10 SI

S3 **Heterozygosity distribution in sorghum F7 mapping population.** (**A**) A rough assessment of the distribution of (excess) heterozygosity of the markers used to genotype the sorghum mapping population shows that most markers display more heterozygosity than expected under Mendelian assumptions of segregation (depicted by a red dashed line). The histogram also shows the average excess heterozygosity (depicted by a purple dashed line) that was used to estimate recombination in the RIL. Quantile-Quantile (Q-Q) plots compare the heterozygosity distributions against (**B**) a uniform distribution and (**C**) a normal distribution. By plotting sample quantiles against theoretical quantiles for the distributions, it can be argued that the excess heterozygosity appears to be more normally distributed than it is uniformly distributed. . . . . . 12 SI

S4 **Heterozygosity on chromosome 2.** Dot plot of the proportion of heterozygous genotypes vs the physical base pair position of markers on chromosome 2 illustrates the variability of heterozygosity observed in regions of the genome. . . . . 13 SI

S5 **Screenshot of spreadsheet containing map estimation results for the sorghum mapping population and simulated data with different models and methods.** This spreadsheet is provided as a .ods and a .xlsx file at `https://github.com/MulletLab/exHet_Supplement` and a description of its results are here in the Supplemental Information text. . . . . 17 SI

LIST OF TABLES

S1 Reports of deviation from expected heterozygosity maintained per generation. The 19.76% from Kong et al. comes from an average of the reported distorted regions across the genome; the total proportion of heterozygosity could not be located in the publication, so this is likely an overestimate. . . . . 5 SI

S2 Reported genetic map sizes for the sorghum BTx623 x IS3620c RIL population used in this study. . . . . 5 SI

Plant	Generation	% Observed	% Expected	Citation
Arabidopsis	F_8	0.42	0.78125	Lister and Dean (1993)
Tomato	F_7	15.0	1.5625	Paran *et al.* (1995)
Maize	F_{10}	1.6	0.1953125	Burr and Burr (1991)
Maize	F_{10}	2.7	0.1953125	Burr and Burr (1991)
Sorghum	F_6	4.69	3.125	Peng *et al.* (1999)
Sorghum	F_5	19.76*	6.25	Kong *et al.* (2013)

Table S1: Reports of deviation from expected heterozygosity maintained per generation. The 19.76% from Kong et al. comes from an average of the reported distorted regions across the genome; the total proportion of heterozygosity could not be located in the publication, so this is likely an overestimate.

Marker #	Reported map size (cM)	Citation	Marker Type
145	1279	Hart *et al.* (2001)	RFLP, SSR
323	1347	Peng *et al.* (1999)	RFLP
466	1406	Bhattramakki *et al.* (2000)	RFLP, SSR
792	1528	Mace *et al.* (2009)	DaRT, RFLP, SSR
2926	1713	Menz *et al.* (2002)	AFLP, RFLP, SSR

Table S2: Reported genetic map sizes for the sorghum BTx623 x IS3620c RIL population used in this study.

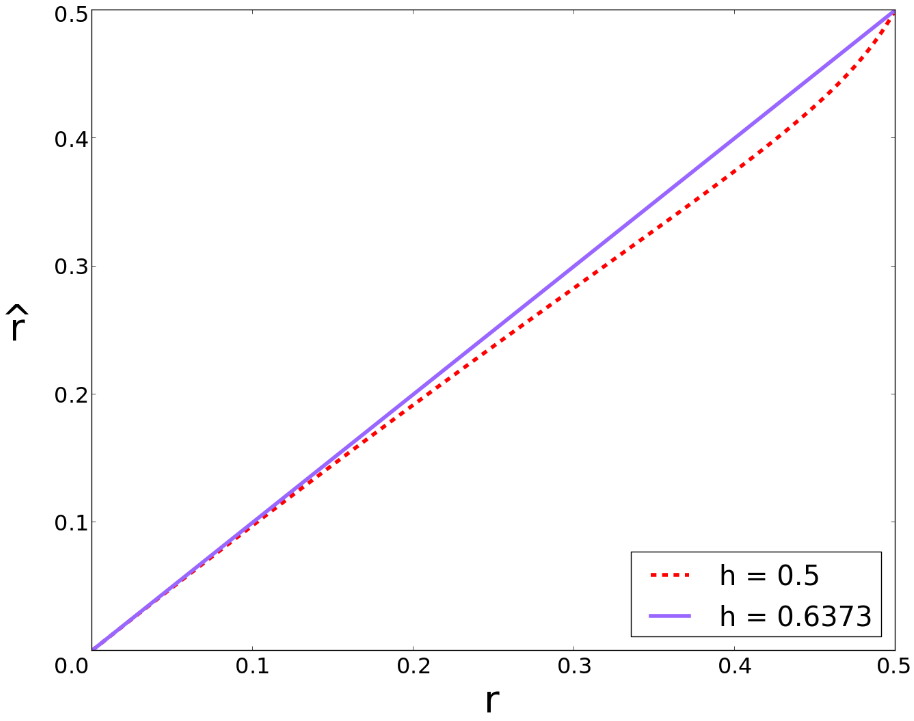

Figure S1: **Estimated recombination fractions, $\hat{r}$, of excess heterozygosity versus Mendelian expectations for $t = 3$.** Recombination fractions estimated from genotype frequencies under Mendelian expectations (h=0.5) versus under modeling a global heterozygosity advantage (h=0.6373) at generation $t = 3$ of a selfing population. This shows that if the population was retaining excess heterozygosity (at a rate of 63.73% each generation as opposed to the Mendelian 50%), then estimating recombination fractions under Mendelian expectations would shrink the map if observed at generation $t = 3$.

SOLVING FOR THE GENERAL SOLUTION, P_{F_T}, IN MATLAB

Given the theory derived for $p_{F_t}{}' = \mathbf{T}p_{F_{t-1}}{}'$ we solved for the general solution of p_{F_t} using MATLAB (2010) and an M-file is provided as a supplemental file to document all variables defined and calculations. The M-file can be found on `https://github.com/MulletLab/exHet_Supplement`, and we also provide it below.

```
% MATLAB M-file to derive the general solution for the probability of
% a marker in a genotype class given a selfing population, Ft, for t
% generations. This is supplemental information for Truong & McCormick
% et al (2014) where we incorporate a heterozygosity zygotic viability term.
%
    % assign variables:
% r is the recombination frequency, and t is the generation interval
 syms r t;
    % h is amount of heterozygosity maintained in each generation and can
be
% parameterized given generation t. That is if H is the amount of
% heterozygosity in an Ft population, then h^(t-1)=H
 syms h;
    % u is the viability of Aa to AA and aa
% solve(h == ((2*u^2)*((1-r)^2+r^2) + (2*2*u*r*(1-r)))/d , u )
 syms u;
u = -(2*h*r - r + ((r^2 - 2*h*r + h)*(2*h*r - 2*r - h + r^2 + 1))^(1/2) -
2*h*r^2 + r^2)/(h + 2*r - 2*h*r + 2*h*r^2 - 2*r^2 - 1);
    % d is a parameter necessary to weigh to u appropriately
 syms d;
d = 2*((1-r)^2)+8*u*r*(1-r)+ 2*(r^2)+ 2*(u^2)*(((1-r)^2)+(r^2);
% Transition probability matrix for 5 classes of genotypes
 T = [
1, 0, (1-h^1)/2, (2*((1-r^1)^2))/d^1, (2*(r^2))/d^1;
0, 1, (1-h^1)/2, (2*(r^2))/d^1, (2*((1-r^1)^2))/d^1;
0, 0, (h^1), (8*u^1*r^1*(1-r^1)^1)/d^1, (8*u^1*r^1*(1-r^1)^1)/d^1;
0, 0, 0, (2*u^2*(1-r^1)^2)/d^1, (2*u^2*r^2)/d^1;
0, 0, 0, (2*u^2*r^2)/d^1, (2*u^2*(1-r^1)^2)/d^1];
```

```
% Take eigenvalues of Transition probability matrix to set up system of
% equations to find the general solution given generation t for all 5
% classes
 eigT = eig(T);
% qit=[
% p(class 1 in generation t);
% p(class 2 in generation t);
% p(class 3 in generation t);
% p(class 4 in generation t);
% p(class 5 in generation t)];
% Initialize probability of class given generation t. For an F1 (t=1)
% from the initial mating of homozygous parents (ie AABB x aabb), all
% individuals in the F1 are of class 4 (ie AaBb in coupling (AB/ab))
 qi1=[0;0;0;1;0];
qi2 = T*qi1;
qi3 = T*qi2;
qi4 = T*qi3;
% bclass = [
% p(class in F1);
% p(class in F2);
% p(class in F3);
% p(class in F4)];
% Set up the frequences directly in F1, F2, F3, and F4 for each
% class
 b1=[qi1(1,1);qi2(1,1);qi3(1,1);qi4(1,1)];
b2=[qi1(2,1);qi2(2,1);qi3(2,1);qi4(2,1)];
b3=[qi1(3,1);qi2(3,1);qi3(3,1);qi4(3,1)];
b4=[qi1(4,1);qi2(4,1);qi3(4,1);qi4(4,1)];
b5=[qi1(5,1);qi2(5,1);qi3(5,1);qi4(5,1)];
% Set up the 4 linear equations (for each generation t=1,2,3,4)
 A=[
eigT(1,1)^1 eigT(2,1)^1 eigT(3,1)^1 eigT(4,1)^1;
eigT(1,1)^2 eigT(2,1)^2 eigT(3,1)^2 eigT(4,1)^2;
eigT(1,1)^3 eigT(2,1)^3 eigT(3,1)^3 eigT(4,1)^3;
eigT(1,1)^4 eigT(2,1)^4 eigT(3,1)^4 eigT(4,1)^4];
% We now have a system of 4 linear equations with 4 unknowns for each
class
% A*[coefficients of general solution]=bclass
```

```
x1=linsolve(A,b1);
x2=linsolve(A,b2);
x3=linsolve(A,b3);
x4=linsolve(A,b4);
x5=linsolve(A,b5);
q_it=[eigT(1,1)^t eigT(2,1)^t eigT(3,1)^t eigT(4,1)^t];
% pclass is the probability of class i (where i=1,2,3,4,5) given
% heterozygosity maintained h, recombination r, and generation t
p1=q_it*x1;
p2=q_it*x2;
p3=q_it*x3;
p4=q_it*x4;
p5=q_it*x5;
```

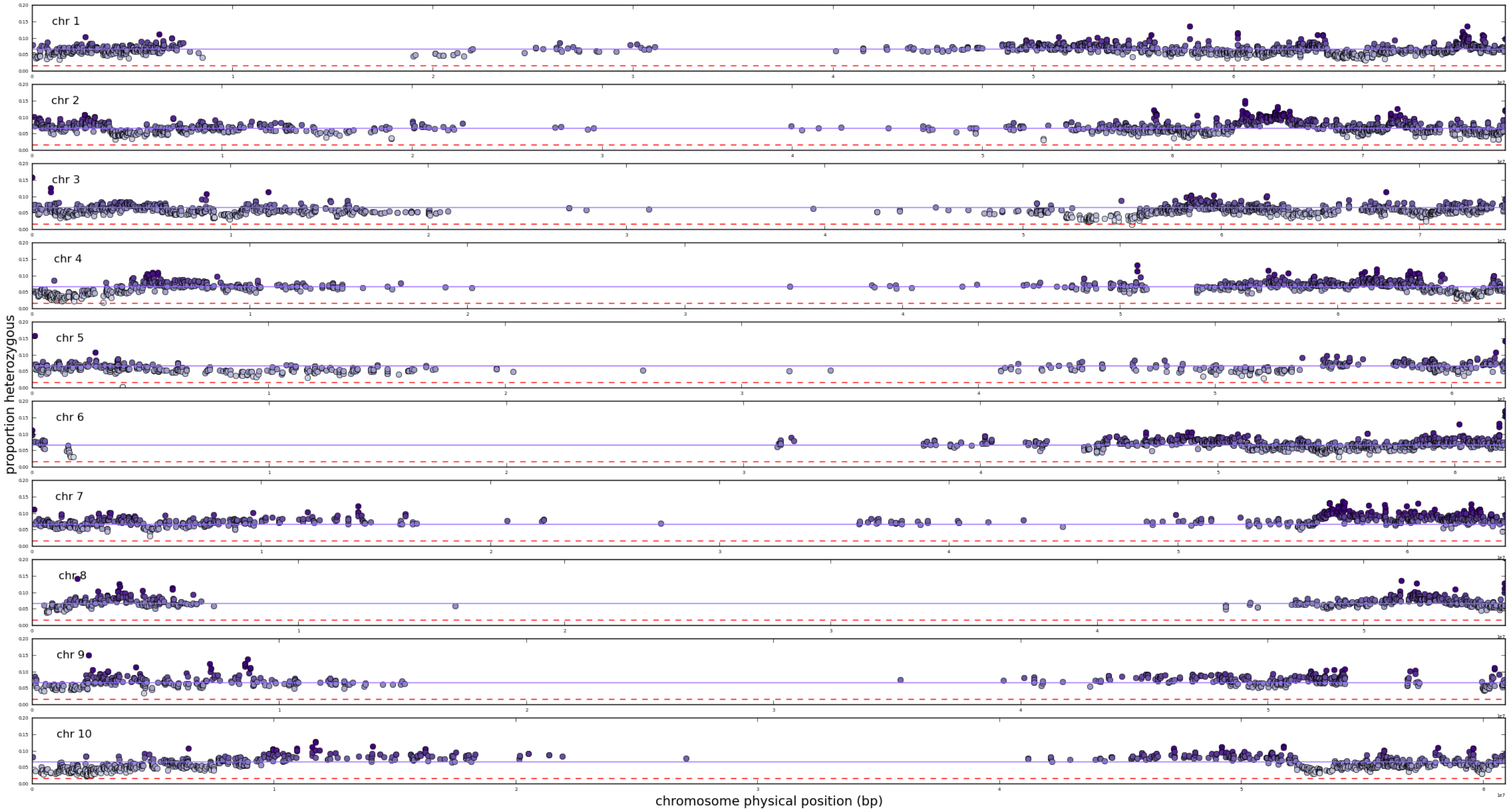

Figure S2: **Heterozygosity landscape.** Dot plot of the proportion of heterozygous genotypes versus the physical base pair position of the 10,081 markers. The coloring of the markers correspond to the percentage of heterozygosity as explained in Figure 3. The Mendelian expected proportion of heterozygosity of an F_7 RIL population is 0.016 and the observed heterozygosity as an average of the BTx623×IS3620c F_7 is 0.067 depicted by a red dashed line and purple solid line, respectively.

PARAMETERIZATION OF THE HETEROZYGOSITY TERM

To generate the sorghum genetic map presented in this study, we modeled a global heterozygosity maintained per generation parameter, h, based on the average heterozygosity observed, H. We briefly discussed the possibility of more local estimations of h, and here we explore the topic in greater detail. Here, we (i) analyze the distribution of heterozygosity, (ii) provide further reasoning to using a global heterozygosity term as well as (iii) present two methods for parameterizing more local fluctuations in h. The first method estimates an h for each linkage group and is implemented as an option in est.rf.exHet(), and the second method derives an h for each marker pair. We also acknowledge an intermediate approach whereby local heterozygosity could be estimated on a regional basis to parameterize a regional h, perhaps with a sliding window.

A global heterozygosity term

Figures S2 and S4 show that there are regions of variable heterozygosity such that groups of markers vary in their proportion of individuals heterozygous relative to the genome-wide average. When modeling recombination fractions under either Mendelian or excess heterozygosity (as done in the paper) it is assumed that genotypes are uniformly distributed. However, we find that the proportion of heterozygous individuals at a marker more closely follows a normal distribution, suggesting that the assumption of a uniform distribution underlying both models may need to be revisited (Figure S3). However, given (i) that the proportions of heterozygosity are greater than those expected under the Mendelian model genome-wide (Figures S2 and S3) and (ii) the precedence for assuming a uniform distribution used when estimating recombination fractions under the Mendelian model, we found the use of a global heterozygosity parameter taken from the average of all markers' genotypes to be a reasonable choice.

Local heterozygosity term for each linkage group, $h_{\text{linkage group \#}}$

An alternative to map estimation using a global heterozygosity term is to estimate h for each linkage group. We implemented this alternative, and in our use cases, employing a local parameterization of the heterozygos-

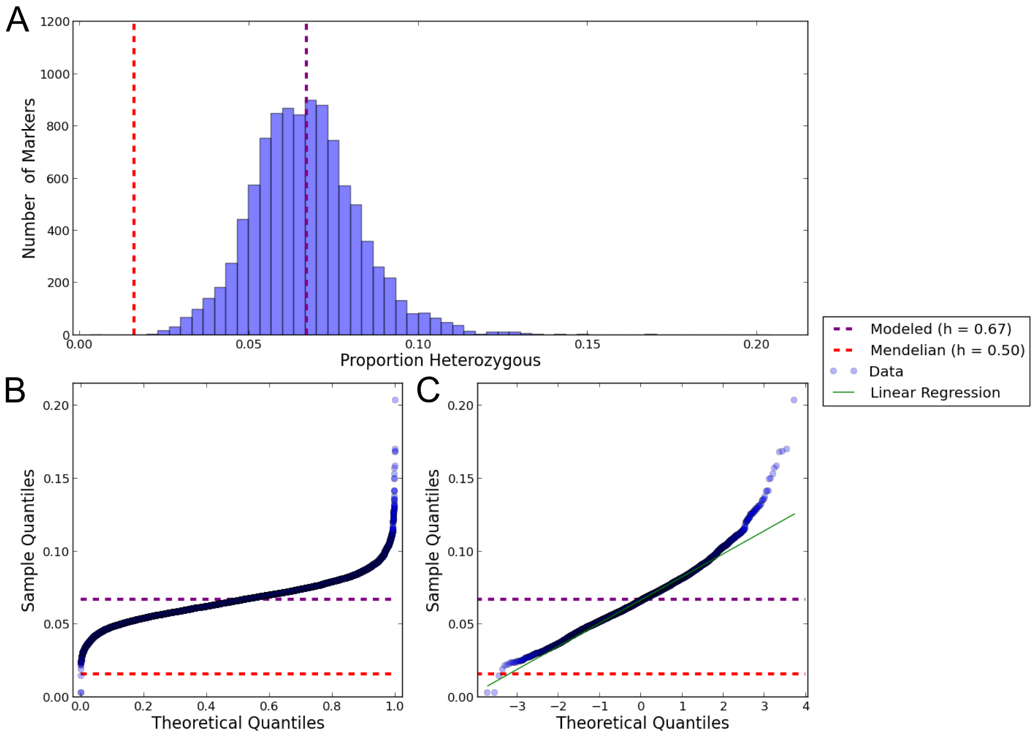

Figure S3: **Heterozygosity distribution in sorghum F7 mapping population.** (**A**) A rough assessment of the distribution of (excess) heterozygosity of the markers used to genotype the sorghum mapping population shows that most markers display more heterozygosity than expected under Mendelian assumptions of segregation (depicted by a red dashed line). The histogram also shows the average excess heterozygosity (depicted by a purple dashed line) that was used to estimate recombination in the RIL. Quantile-Quantile (Q-Q) plots compare the heterozygosity distributions against (**B**) a uniform distribution and (**C**) a normal distribution. By plotting sample quantiles against theoretical quantiles for the distributions, it can be argued that the excess heterozygosity appears to be more normally distributed than it is uniformly distributed.

ity based on linkage groups gave similar results to the global heterozygosity parameterization (see Figure S5 and spreadsheet provided at `https://github.com/MulletLab/exHet_Supplement`). Calculating recombination

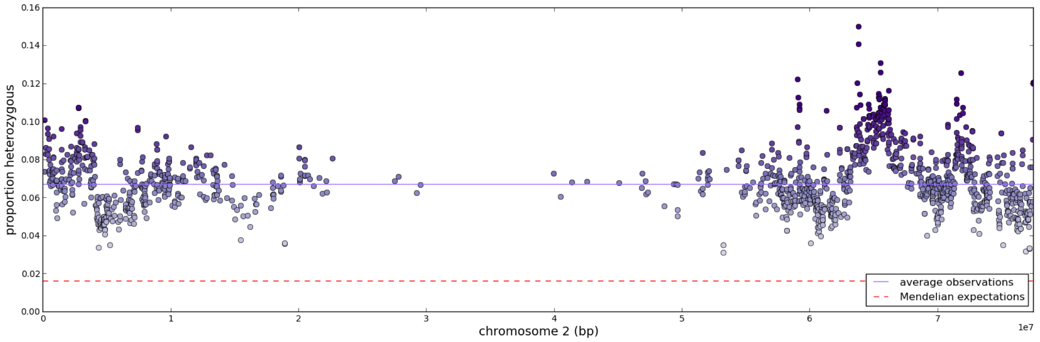

Figure S4: **Heterozygosity on chromosome 2.** Dot plot of the proportion of heterozygous genotypes vs the physical base pair position of markers on chromosome 2 illustrates the variability of heterozygosity observed in regions of the genome.

fractions with local heterozygosity based on linkage groups can be used by invoking est.rf.exHet(hetByLinkageGroup=TRUE). An example of how to call the function used to parameterize h by linkage groups is provided in the example code at `https://github.com/MulletLab/exHet_Supplement`.

The derivation for a local heterozygosity term for each marker, $h_{\mathbf{marker}}$

Here we briefly discuss a general solution, ${p_{F_t}}' = \mathbf{T}{p_{F_{t-1}}}'$, to be solved for in order to incorporate differential heterozygosity for each marker. While we derive it here, we chose not to use it and did not implement it due to the pitfalls associated with overfitting data.

This follows the theory described in the paper such that we will build the transition probability matrix and then solve for the general solution of p_{F_t}. First we will redefine our genotype classes. We are going to treat different markers with differential heterozygosity terms, so it would be nice to split the single heterozygote class (enumerated class 3 in the paper) to class 3_α and class 3_β. Such that now we have

$$p_{F_t} = \begin{pmatrix} p(\text{class 1}) \\ p(\text{class 2}) \\ p(\text{class } 3_\alpha) \\ p(\text{class } 3_\beta) \\ p(\text{class 4}) \\ p(\text{class 5}) \end{pmatrix}_t = \overset{p(\text{genotypes})}{\begin{pmatrix} p(\frac{\text{AB}}{\text{AB}}) + p(\frac{\text{ab}}{\text{ab}}) \\ p(\frac{\text{Ab}}{\text{Ab}}) + p(\frac{\text{aB}}{\text{aB}}) \\ p(\frac{\text{AB}}{\text{aB}}) + p(\frac{\text{Ab}}{\text{ab}}) \\ p(\frac{\text{AB}}{\text{Ab}}) + p(\frac{\text{aB}}{\text{ab}}) \\ p(\frac{\text{AB}}{\text{ab}}) \\ p(\frac{\text{Ab}}{\text{aB}}) \end{pmatrix}_t}$$

class 1 and class 2: The transition from class 1 and class 2 in generation t to generation $t+1$ are fixed.

class 3: The transition from class 3 in generation t to generation $t+1$ will take into consideration only the segregation of one marker that is heterozygote in generation t as the other marker will be homozygote and thus fixed in any subsequent generation after t.

If H_{α,F_t} proportion of heterozygosity observed in marker α for an F_t family and we assume that the amount of heterozygosity maintained in marker α, h_α, each generation prior to generation t is the same, then we can solve for h_α through the following relationship ${h_\alpha}^{t-1} = H_{\alpha,F_t}$. h_α will be modeled into the transition probability matrix as a modifier of expected segregation. To do so, we can treat marker α's genotypes (zygotes) with differential viability (expectation to be observed in the next generation). Define the amount of heterozygosity maintained at marker α as h_α (parameterized from data as shown above) through selfing. Then our expected segregation ratio for $AA : Aa : aa$ is

$$\frac{1-h_\alpha}{2} : h_\alpha : \frac{1-h_\alpha}{2}.$$

Notice that under the assumption of Mendelian segregation, $h_\alpha = 1/2$ and the expected Mendelian segregation would then be the familiar $1:2:1$. The same model is true for marker β.

class 4 and 5: The transition from class 4 and 5 in generation t to generation $t+1$ will take into consideration both the segregation of two markers that are heterozygous at generation t and the recombination frequency between the two markers.

Similar to treatment of heterozygosity for one marker, we now have a heterozygosity term for both marker α, h_α, and marker β, h_β. Given two heterozygosity terms (one for each marker), we can parameterize both h_{marker}'s for each pair of markers in genetic map construction. Now, in the context of zygotic differential viability, assume that

1. the viability of genotype Aa relative to AA or aa is u_α (dependent on h_α)
2. the viability of genotype Bb relative to BB or bb is u_β (dependent on h_β)

such that the expected segregation is now

$$\mathbf{prob}(\text{genotype}_{\ t} \,|\, \text{class 4}_{\ t-1}) = \begin{array}{c} \\ \text{BB} \\ \text{Bb} \\ \text{bb} \end{array} \begin{array}{c} \begin{array}{ccc} \text{AA} & \text{Aa} & \text{aa} \end{array} \\ \begin{pmatrix} \frac{1}{d}(1-r)^2 & \frac{2u_\alpha}{d}r(1-r) & \frac{1}{d}r^2 \\ \frac{2u_\beta}{d}r(1-r) & \frac{2u_\alpha u_\beta}{d}[r^2+(1-r)^2)] & \frac{2u_\beta}{d}r(1-r) \\ \frac{1}{d}r^2 & \frac{2u_\alpha}{d}r(1-r) & \frac{1}{d}(1-r)^2 \end{pmatrix} \end{array}$$

where $d = 2(1-r)^2 + 4u_\alpha r(1-r) + 4u_\beta r(1-r) + 2r^2 + 2u_\alpha u_\beta[(1-r)^2 + r^2]$. Then, the amount of heterozygosity retained in generation t for a marker pair of either class 4 or 5[1] in the previous generation $t-1$ should satisfy

$$h = \frac{1}{2}\text{prob(AaBB)} + \frac{1}{2}\text{prob(AABb)} + \text{prob(AaBb)} + \frac{1}{2}\text{prob(aaBb)} + \frac{1}{2}\text{prob(Aabb)}$$

such that given data H_{α,F_t}, H_{β,F_t}, and t we can calculate

$$h_{\text{marker}} = e^{\frac{\ln(H_{\text{marker}},F_t)}{t-1}}.$$

Furthermore, given data r for each marker pair we can subsequently calculate u_α, u_β and d . **Transition probability matrix**: Incorporating the transition from a class # to other classes (from the previous sections) in every generation, we now have a transition probability matrix,

$$\mathbf{T} = \begin{array}{c} \\ \text{class 1} \\ \text{class 2} \\ \text{class } 3_\alpha \\ \text{class } 3_\beta \\ \text{class 4} \\ \text{class 5} \end{array} \begin{array}{c} \begin{array}{cccccc} \text{class 1} & \text{class 2} & \text{class } 3_\alpha & \text{class } 3_\beta & \text{class 4} & \text{class 5} \end{array} \\ \begin{pmatrix} 1 & 0 & \frac{1-h_\alpha}{2} & \frac{1-h_\beta}{2} & \frac{2(1-r)^2}{d} & \frac{2r^2}{d} \\ 0 & 1 & \frac{1-h_\alpha}{2} & \frac{1-h_\beta}{2} & \frac{2r^2}{d} & \frac{2(1-r)^2}{d} \\ 0 & 0 & h_\alpha & 0 & \frac{4u_\alpha r(1-r)}{d} & \frac{4u_\alpha r(1-r)}{d} \\ 0 & 0 & 0 & h_\beta & \frac{4u_\beta r(1-r)}{d} & \frac{4u_\beta r(1-r)}{d} \\ 0 & 0 & 0 & 0 & \frac{2u_\alpha u_\beta(1-r)^2}{d} & \frac{2u_\alpha u_\beta r^2}{d} \\ 0 & 0 & 0 & 0 & \frac{2u_\alpha u_\beta r^2}{d} & \frac{2u_\alpha u_\beta(1-r)^2}{d} \end{pmatrix} \end{array}$$

[1]A probability matrix of all genotypes created after selfing of class 5 would look similar except for the exchange of rows 1 and 3.

With $\mathbf{T}$ defined, we can solve for the general solution of p_{F_t} dependent on t, r, and H_{F_t} for every marker pair. Solving for the general solution here is conceptually similar to the process described for the global heterozygosity term and described in the M-file in section 1.

GENETIC MAP ESTIMATIONS OF SIMULATED DATASETS WITH EXCESS HETEROZYGOSITY

	A	B	D	F	H	I	K	M	O	Q
1	BTx623 x IS3620c – short double crossovers (SDCO) set to missing									
2				F7 est.rf.exHet(h=0.6373)	F7 est.rf.exHet(hetByLinkageGroup=TRUE)		F7 est.rf.exHet(h=0.5)	F7 est.rf()	F7 est.map(errorprob=0.01)	RIL est.map(errorprob=0.01)
3	Linkage Group	Marker #		cM distance	estimated h	cm distance	cM distance	cM distance	cM distance	cM distance
4	1	1418		177	0.6350	177.6	206.7	206.7	203.0	146.5
5	2	1398		185.9	0.6399	185.3	213.4	213.4	211.4	151.5
6	3	1495		179.2	0.6234	182.6	208.6	208.6	207.2	143.9
7	4	1039		146.2	0.6350	146.6	169.2	169.2	172.4	128.3
8	5	663		109.4	0.6273	110.8	126.2	126.2	132.8	98.1
9	6	1007		117.6	0.6419	116.8	135.7	135.7	145.5	105.5
10	7	804		113	0.6551	110.6	127.5	127.5	133.5	103.3
11	8	528		101	0.6451	100.0	114.7	114.7	122.6	90.3
12	9	641		118.7	0.6441	117.6	135.3	135.3	138.1	101.9
13	10	1088		142.8	0.6291	144.4	166.6	166.6	162.9	107.4
14	Total	10081		1390.6		1392.3	1603.8	1603.8	1629.3	1176.7
15										
16	BTx623 x IS3620c - with short double crossovers (SDCO)									
17				F7 est.rf.exHet(h=0.6373)	F7 est.rf.exHet(hetByLinkageGroup=TRUE)		F7 est.rf.exHet(h=0.5)	F7 est.rf()	F7 est.map(errorprob=0.01)	RIL est.map(errorprob=0.01)
18	Linkage Group	Marker #		cM distance	estimated h	cm distance	cM distance	cM distance	cM distance	cM distance
19	1	1418		468.7	0.6467	463.1	534.7	534.7	452.9	160.1273
20	2	1398		461.4	0.6522	453.1	521.6	521.6	435.5	158.2769
21	3	1495		498.2	0.6381	497.7	571.3	571.3	481.7	151.9861
22	4	1039		370.8	0.6476	366.1	421.6	421.6	362.7	134.1605
23	5	663		241.4	0.6407	240.3	275.8	275.8	247.4	105.2321
24	6	1007		322.5	0.6544	315.8	364.8	364.8	312.1	113.5757
25	7	804		269.2	0.6650	260.9	299.2	299.2	258.8	110.3455
26	8	528		205.5	0.6559	201.0	230.5	230.5	217.4	95.44168
27	9	641		251.2	0.6556	245.8	281.7	281.7	251.3	107.4022
28	10	1088		371.9	0.6426	369.3	426.4	426.4	365.3	111.5893
29	Total	10081		3460.9		3413.2	3927.6	3927.6	3385.2	1248.137
30										
31	Simulated (n=1000, t=7, h=0.6373) – No errors, no missing data									
32			Simulated Distance	F7 est.rf.exHet(h=0.6373)	F7 est.rf.exHet(hetByLinkageGroup=TRUE)		F7 est.rf.exHet(h=0.5)	F7 est.rf()	F7 est.map(errorprob=0.01)	RIL est.map(errorprob=0.01)
33	Linkage Group	Marker #	cM distance	cM distance	estimated h	cm distance	cM distance	cM distance	cM distance	cM distance
34	1	1000	200	204.9	0.6346	205.6	236.0	236.0	234.0	227.8
35										
36	Simulated (n=1000, t=7, h=0.6373) – Errors (1%) and missing data (5%) (i.e. With SDCOs)									
37			Simulated Distance	F7 est.rf.exHet(h=0.6373)	F7 est.rf.exHet(hetByLinkageGroup=TRUE)		F7 est.rf.exHet(h=0.5)	F7 est.rf()	F7 est.map(errorprob=0.01)	RIL est.map(errorprob=0.01)
38	Linkage Group	Marker #	cM distance	cM distance	estimated h	cm distance	cM distance	cM distance	cM distance	cM distance
39	1	1000	200	742.8	0.6375	742.6	854.2	854.2	240.2	237.6
40										
41	Simulated (n=1000, t=7, h=0.6373) – Errors (1%) and Missing Data (5%) - SDCOs set to missing									
42			Simulated Distance	F7 est.rf.exHet(h=0.6373)	F7 est.rf.exHet(hetByLinkageGroup=TRUE)		F7 est.rf.exHet(h=0.5)	F7 est.rf()	F7 est.map(errorprob=0.01)	RIL est.map(errorprob=0.01)
43	Linkage Group	Marker #	cM distance	cM distance	estimated h	cm distance	cM distance	cM distance	cM distance	cM distance
44	1	1000	200	196.3	0.6340	197.1	226.3	226.3	231.6	224.5

Figure S5: **Screenshot of spreadsheet containing map estimation results for the sorghum mapping population and simulated data with different models and methods.** This spreadsheet is provided as a .ods and a .xlsx file at `https://github.com/MulletLab/exHet_Supplement` and a description of its results are here in the Supplemental Information text.

This section describes a simulation study performed to demonstrate the effect of accounting for excess heterozygosity in the genetic model. Figure S5 and the associated spreadsheet found at `https://github.com/MulletLab/exHet_Supplement` provide the results of estimating genetic maps for the sorghum mapping population and the simulated data using different models and methods. The following factors were considered:

1. Dataset
   (a) BTx623 x IS3620c with tight double recombinations removed
   (b) BTx623 x IS3620c without tight double recombinations removed
   (c) Simulated data generated under conditions of excess heterozygosity

   (d) Simulated data generated under conditions of excess heterozygosity with 1% error rate and 5% missing data
   (e) Simulated data generated under conditions of excess heterozygosity with 1% error rate and 5% missing data with tight double recombinations removed

2. Method
   (a) Pairwise estimation using est.rf() or est.rf.exHet(), where est.rf.exHet(h = 0.5) is equal to est.rf()
   (b) Multipoint estimation with a hidden Markov model using est.map() and a 1% error probability

3. Model
   (a) Mendelian model ($h = 0.5$)
   (b) Derived heterozygosity model, global h ($h = 0.6373$)
   (c) Derive heterozygosity model, local h by linkage group (hetByLinkageGroup=TRUE)

4. Generation Interval
   (a) F_7
   (b) Fixed RIL ($t \to \infty$)

Tight double recombinations

Tight double recombinations, also referred to as short double crossovers (SDCOs) in the provided code and results, are most often treated as genotyping errors. In the sorghum mapping population dataset used in this paper, setting short double crossovers smaller than 2 cM to missing removed 1.1% of the genotypes (37,299 out of 3,407,539). When simulating genotyping errors, we used a 1% error rate. The method we used to remove short double crossovers was sufficient to compensate for a 1% error rate in the simulated dataset, such that both pairwise estimation (i.e. using est.rf.exHet()) and multipoint estimation using the HMM (i.e. using est.map()) provided comparable results between (i) the simulated data with 1% error rate with tight double recombinations removed and (ii) the simulated data without error. The method we used to remove tight double recombinations is provided as a Python script at `https://github.com/MulletLab/exHet_Supplement`.

Error probability in the hidden Markov model

The results from map estimation using a hidden Markov model (HMM) as implemented in R/qtl's est.map() function on the simulated datasets and the sorghum mapping population showed that the HMM methodology handled the error rate effectively in the simulated data, compensating for the 1% error rate in the simulated data and yielding very similar results whether or not tight double recombinations were removed (though still giving expanded maps since the underlying genetic model assumed $h = 0.5$). However, the HMM multipoint methodology yields very different results between the sorghum mapping dataset with and without tight double recombinations removed. Both the multipoint and the pairwise give grossly inflated maps if tight double recombinations are not removed ($>$ 3000 cM), and give comparable results once tight double crossovers are removed (around 1600 cM). This suggests that the random errors introduced in the simulation were not representative of the errors in sorghum mapping dataset.

F7 versus fixed RIL ($t \to \infty$)

Removal of all heterozygous genotypes (e.g. treating the map as a fixed RIL) reduces the map size for both the simulated and real datasets (under both pairwise and multipoint methods). This is expected since the removal of heterozygous genotypes effectively removes recombination events; in the case of our real dataset this omits 6.7% of the genotypes, or 224,437 genotype calls. For the simulated dataset, the estimated map is still larger than the simulated linkage group since the underlying genetic model does not account for the excess heterozygosity. Unlike the simulated dataset, the multipoint method (i.e. est.map()) yields very different results between treating the real data as an F7 and as a fixed RIL, especially if tight double recombinants are not removed. We suspect that this may be a consequence of the error modeled by the HMM not being representative of how the error exists in the read data.

LITERATURE CITED

Bhattramakki, D., J. Dong, A. K. Chhabra, and G. E. Hart, 2000 An integrated SSR and RFLP linkage map of *Sorghum bicolor* (L.) moench. Genome **43**: 988–1002.

Burr, B., and F. A. Burr, 1991 Recombinant inbreds for molecular mapping in maize: theoretical and practical considerations. Trends in Genetics **7**: 55–60.

Hart, G. E., K. F. Schertz, Y. Peng, and N. H. Syed, 2001 Genetic mapping of *Sorghum bicolor* (L.) Moench QTLs that control variation in tillering and other morphological characters. Theoretical and Applied Genetics **103**: 1232–1242.

Kong, W., H. Jin, C. D. Franks, C. Kim, R. Bandopadhyay, *et al.*, 2013 Genetic analysis of recombinant inbred lines for *Sorghum bicolor* × *Sorghum propinquum*. G3: Genes— Genomes— Genetics **3**: 101–108.

Lister, C., and C. Dean, 1993 Recombinant inbred lines for mapping RFLP and phenotypic markers in *Arabidopsis thaliana*. The Plant Journal **4**: 745–750.

Mace, E. S., J.-F. Rami, S. Bouchet, P. E. Klein, R. R. Klein, *et al.*, 2009 A consensus genetic map of sorghum that integrates multiple component maps and high-throughput Diversity Array Technology (DArT) markers. BMC Plant Biology **9**: 13.

MATLAB, 2010 *version 7.10.0 (R2010a)*. The MathWorks Inc., Natick, Massachusetts.

Menz, M., R. Klein, J. Mullet, J. Obert, N. Unruh, *et al.*, 2002 A high-density genetic map of *Sorghum bicolor* (L.) Moench based on 2926 AFLP, RFLP and SSR markers. Plant Molecular Biology **48**: 483–499.

Paran, I., I. Goldman, S. Tanksley, and D. Zamir, 1995 Recombinant inbred lines for genetic mapping in tomato. Theoretical and Applied Genetics **90**: 542–548.

Peng, Y., K. F. Schertz, S. Cartinhour, and G. E. Hart, 1999 Comparative genome mapping of *Sorghum bicolor* (L.) Moench using an RFLP map constructed in a population of recombinant inbred lines. Plant Breeding **118**: 225–235.
