# Supplementary material for: Resolution of Genetic Map Expansion Caused by Excess Heterozygosity in Plant Recombinant Inbred Populations
Source: G3 (Bethesda). 2014 Aug 15;4(10):1963–9. doi: 10.1534/g3.114.012468 (PMC4199702; doi:10.1534/g3.114.012468)
Supplement: Supporting Information [file supp_g3.114.012468_FigureS1.pdf]

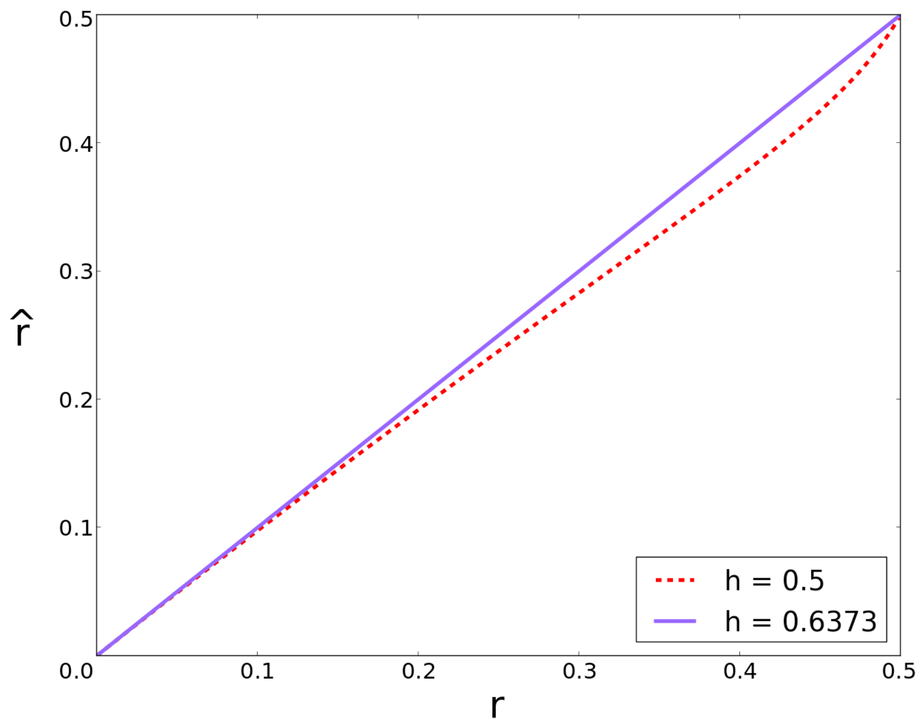

Figure S1: **Estimated recombination fractions, $\hat{r}$, of excess heterozygosity versus Mendelian expectations for $t = 3$.** Recombination fractions estimated from genotype frequencies under Mendelian expectations (h=0.5) versus under modeling a global heterozygosity advantage (h=0.6373) at generation $t = 3$ of a selfing population. This shows that if the population was retaining excess heterozygosity (at a rate of 63.73% each generation as opposed to the Mendelian 50%), then estimating recombination fractions under Mendelian expectations would shrink the map if observed at generation $t = 3$.
