# Supplementary material for: Resolution of Genetic Map Expansion Caused by Excess Heterozygosity in Plant Recombinant Inbred Populations
Source: G3 (Bethesda). 2014 Aug 15;4(10):1963–9. doi: 10.1534/g3.114.012468 (PMC4199702; doi:10.1534/g3.114.012468)
Supplement: Supporting Information [file supp_g3.114.012468_FigureS2.pdf]

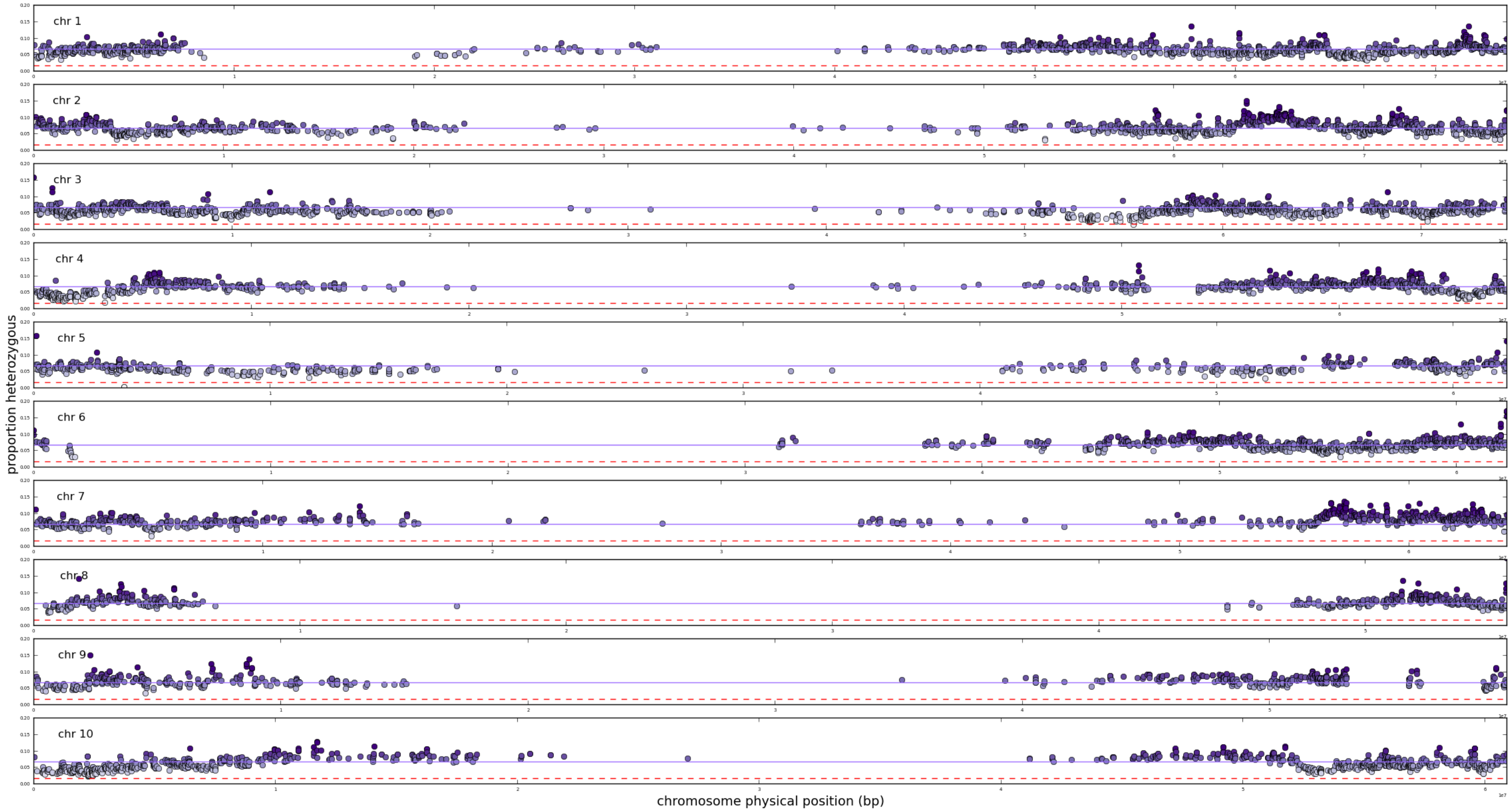

Figure S2: **Heterozygosity landscape.** Dot plot of the proportion of heterozygous genotypes versus the physical base pair position of the 10,081 markers. The coloring of the markers correspond to the percentage of heterozygosity as explained in Figure 3. The Mendelian expected proportion of heterozygosity of an F_7 RIL population is 0.016 and the observed heterozygosity as an average of the BTx623×IS3620c F_7 is 0.067 depicted by a red dashed line and purple solid line, respectively.
