# Supplementary figures and images for: Resolution of Genetic Map Expansion Caused by Excess Heterozygosity in Plant Recombinant Inbred Populations
Source: G3 (Bethesda). 2014 Aug 15;4(10):1963–9. doi: 10.1534/g3.114.012468 (PMC4199702; doi:10.1534/g3.114.012468)

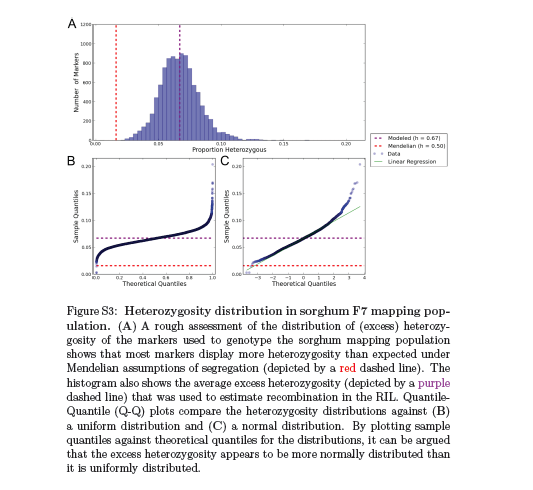

Supplement: Supporting Information [file supp_g3.114.012468_FigureS3.tif]

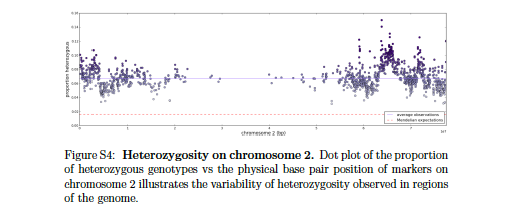

Supplement: Supporting Information [file supp_g3.114.012468_FigureS4.tif]

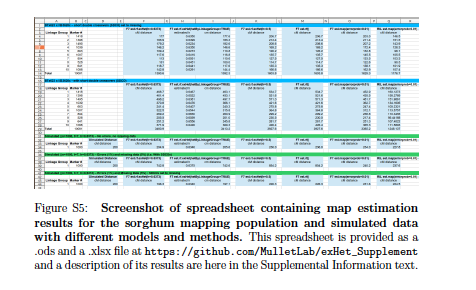

Supplement: Supporting Information [file supp_g3.114.012468_FigureS5.tif]

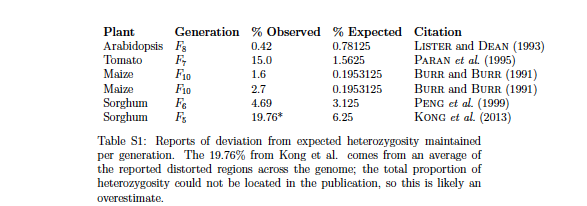

Supplement: Supporting Information [file supp_g3.114.012468_TableS1.tif]

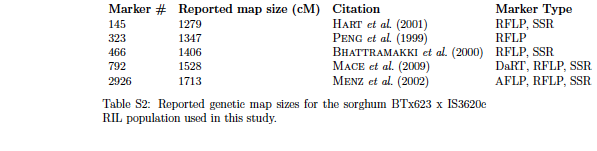

Supplement: Supporting Information [file supp_g3.114.012468_TableS2.tif]
